# Nitrate of Silver

**Published:** 1891-10

**Authors:** 


### NITRATE OF SILVER.—STEBBINS.

We generally permit papers published in the Journal to go to the readers without notice, but the one prepared by Dr. Stebbins, in this number, giving his experience with the treatment of caries in deciduous teeth, is so remarkable as to demand special attention. If his results are sustained by others it must be regarded as a positive advance in the treatment of these teeth, and opens up possibilities in other directions difficult now to realize.

We had the pleasure of examining cases at Saratoga treated with this agent at different periods, the longest six years, and, while exposed all that time to the action of the secretions of the mouth, they gave no evidence of destructive action.

Dr. Stebbins has taken the truly scientific course in waiting and experimenting for six years before giving this to the profession.

